# Cognitive Screening with the Italian International HIV Dementia Scale in People Living with HIV: A Cross-Sectional Study in the cART Era

**DOI:** 10.3390/idr17040095

**Published:** 2025-08-06

**Authors:** Maristella Belfiori, Francesco Salis, Sergio Angioni, Claudia Bonalumi, Diva Cabeccia, Camilla Onnis, Nicola Pirisi, Francesco Ortu, Paola Piano, Stefano Del Giacco, Antonella Mandas

**Affiliations:** 1 Department of Medical Sciences and Public Health, University of Cagliari, 09042 Cagliari, Italy; francesco-salis@tiscali.it (F.S.); seangioni@gmail.com (S.A.); claudia.mt.bonalumi@gmail.com (C.B.); onniscamilla01@gmail.com (C.O.); nicpir95@gmail.com (N.P.); delgiacco@unica.it (S.D.G.); amandas@unica.it (A.M.); 2 Department of Biomedical Sciences, University of Cagliari, 09042 Cagliari, Italy; 3 Department of Medicine, Surgery and Pharmacy, University of Sassari, 07100 Sassari, Italy; cabecciadiva@gmail.com; 4 Department of Internal Medicine, University Hospital “Azienda Ospedaliero-Universitaria” of Cagliari, 09042 Cagliari, Italy; fortu@aoucagliari.it (F.O.); ppiano@aoucagliari.it (P.P.)

**Keywords:** HIV-associated neurocognitive disorders (HANDs), International HIV Dementia Scale (IHDS), cognitive screening tools

## Abstract

**Background**: HIV-associated neurocognitive disorders (HANDs) continue to be a significant concern, despite the advancements in prognosis achieved through Combination Antiretroviral Therapy (cART). Neuropsychological assessment, recommended by international guidelines for HANDs diagnosis, can be resource-intensive. Brief screening tools, like the International HIV Dementia Scale (IHDS) and the Montreal Cognitive Assessment (MoCA), are crucial in facilitating initial evaluations. This study aims to assess the Italian IHDS (IHDS-IT) and evaluate its sensitivity and specificity in detecting cognitive impairment in HIV patients. **Methods**: This cross-sectional study involved 294 patients aged ≥30 years, evaluated at the Immunology Unit of the University of Cagliari. Cognitive function was assessed using the MoCA and IHDS. Laboratory parameters, such as CD4 nadir, current CD4 count, and HIV-RNA levels, were also collected. Statistical analyses included Spearman’s correlation, Receiver Operating Characteristic analysis, and the Youden J statistic to identify the optimal IHDS-IT cut-off for cognitive impairment detection. **Results**: The IHDS and MoCA scores showed a moderate positive correlation (Spearman’s rho = 0.411, *p* < 0.0001). ROC analysis identified an IHDS-IT cut-off of ≤9, yielding an Area Under the Curve (AUC) of 0.76, sensitivity of 71.7%, and specificity of 67.2%. At this threshold, 73.1% of patients with MoCA scores below 23 also presented abnormal IHDS scores, highlighting the complementary utility of both cognitive assessment instruments. **Conclusions**: The IHDS-IT exhibited fair diagnostic accuracy for intercepting cognitive impairment, with a lower optimal cut-off than previously reported. The observed differences may reflect this study cohort’s demographic and clinical characteristics, including advanced age and long-lasting HIV infection. Further, longitudinal studies are necessary to validate these findings and to confirm the proposed IHDS cut-off over extended periods.

## 1. Introduction

In Italy, as well as worldwide, the introduction of Combination Antiretroviral Therapy (cART) has led to a remarkable change in the prognosis of human immunodeficiency virus (HIV) infection and its most advanced stage, Acquired Immunodeficiency Syndrome (AIDS) [1,2,3,4]. However, this increased life expectancy has been accompanied by a growing prevalence of HIV-associated neurocognitive disorders (HANDs) [5,6,7]. Indeed, HIV targets the brain, which represents the second most frequently infected organ after the lungs, because neural cells and microglia of the Central Nervous System (CNS) express chemokine receptors such as CXCR4, which facilitate viral entry [8,9,10]. Infection may occur through both direct mechanisms, via neurotoxic viral proteins, and indirect mechanisms involving neuroinflammation [8,9].

A recent review of 123 studies involving 35,513 people living with HIV/AIDS (PLWHA) reported an overall HANDs prevalence of 42.6%. The authors highlighted differences in prevalence among the three HANDs subtypes, defined by the Frascati criteria: the milder forms, Asymptomatic Neurocognitive Impairment and Mild Neurocognitive Disorder, had prevalences of 23.5% and 13.3%, respectively; in contrast, the most severe form, HIV-associated dementia (HAD), was less common at 5.0% [11]. In the post-cART era, alongside the increased prevalence of milder HANDs forms, the proportion of older PLWHA is rising [12,13,14,15,16,17]. According to the Global AIDS Update (2024), in some high-income countries, nearly half of adults receiving HIV care are aged 50 years or older, and approximately one in 11 is aged 65 or above [18].

Although cART has significantly reduced HIV-associated morbidity and mortality, making HIV infection a chronic manageable disease, HANDs continues to be prevalent [19,20,21]. The impact of antiretroviral therapy on cognitive function remains unclear. Some studies have shown that antiretroviral drugs with high CNS penetration effectiveness (CPE) scores, such as zidovudine, nevirapine, and efavirenz, are associated with improved neurocognitive performance, especially in patients with poor physical condition and immune status [22,23]. Conversely, other research has found that cognitive decline may persist even with effective cART [24,25]. Despite the conflicting positions among researchers regarding the role of antiretroviral therapy on cognitive impairment, in the cART era, the clinical features and the progression of HANDs have changed. Specifically, the clinical phenotype has shifted from a predominantly subcortical disorder, characterized by motor dysfunction, memory impairment, and extrapyramidal signs (e.g., bradykinesia, rigidity, and tremor), to a mixed cortical–subcortical pattern, now typically marked by deficits in executive function and working memory [26].

For the HANDs diagnosis, international guidelines recommend a comprehensive neuropsychological assessment, based on Frascati criteria, which evaluates several cognitive domains [27]. This procedure can be both resource-intensive and expensive, necessitating highly trained personnel. In this context, brief cognitive screening instruments are particularly valuable due to their ease of use and feasibility for administration by healthcare providers. These screening tools are designed to identify individuals who may require a more comprehensive neuropsychological assessment. Among the various screening tools available, the Montreal Cognitive Assessment (MoCA) and the International HIV Dementia Scale (IHDS) are frequently utilized. The Italian Society for Infectious and Tropical Diseases endorses the MoCA for its efficacy in detecting milder forms of HANDs [28]. Conversely, the IHDS is recognized for its considerable sensitivity in identifying subcortical deficits commonly associated with HANDs [29,30,31]. The IHDS was developed as a screening tool suitable for diverse cultural, linguistic, and educational backgrounds. However, normative data for the IHDS in the Italian population are currently lacking. This study aims to assess the Italian version of the IHDS (IHDS-IT) and to evaluate its sensitivity and specificity in intercepting symptoms of cognitive impairment in a cohort of HIV patients.

## 2. Materials and Methods

### 2.1. Design of This Study

This study was designed as a cross-sectional analysis involving 294 PLWHA evaluated at the Immunology Unit of the University of Cagliari, Italy. Participants were included if they met the following criteria: age of 30 years or older, confirmed HIV infection, good functional status defined as the ability to perform both basic and instrumental activities of daily living independently (assessed via clinical interview using the Katz and Lawton scales) [32,33], no prior diagnosis of dementia, normal serum levels of vitamin B12, vitamin B9, thyroid-stimulating hormone, and free thyroxine, provided written informed consent, and underwent both immune–virological assessment and cognitive screening tests. Patients were excluded only if they had incomplete clinical or laboratory data.

### 2.2. Laboratory Examinations

The following laboratory plasma parameters were collected for each patient: CD4 nadir, defined as the lowest level of CD4+ T lymphocytes recorded during the patient’s history of HIV infection, indicative of the extent of immune system compromise; HIV-RNA zenith, representing the peak viral load measured in copies/μL during HIV infection; current CD4 count, reflecting the current level of CD4+ T cells, which is crucial for monitoring immune function and response to antiretroviral therapy; CD4/CD8 ratio, considered a marker of immune balance, where a low ratio is often associated with immune dysregulation; current HIV-RNA levels, representing the quantitative measurement of viral replication at the time of this study, essential for evaluating the effectiveness of cART.

### 2.3. Cognitive Screening Tools

Cognitive function was assessed using the following screening tools:

- MoCA, a 30-point screening tool widely used to assess multiple cognitive domains [34]. The MoCA Total Score is adjusted for education, applying a 1-point correction for individuals with ≤12 years of schooling. Although scores ≥26 are generally considered within the normal range, we adopted a cut-off of 23 to define cognitive impairment [34,35]. This threshold was chosen based on a recent systematic review conducted in HIV-positive individuals [35], identifying this threshold as optimal for balancing sensitivity and specificity. While some Italian studies have proposed a cut-off of 22 [36,37], these findings were either based on HIV-negative populations or smaller, less representative HIV-positive cohorts.
- IHDS, a culturally adapted version of the HIV Dementia Scale developed for use in non-English-speaking populations. It replaces tasks such as antisaccadic eye movement error, timed alphabet writing, and cube copy items with more culture-free tests of motor and psychomotor speed [38,39]. The test evaluates motor speed, psychomotor coordination, and memory recall, with scores ranging from 0 to 12. A cut-off score of 10 or below is suggestive of neurocognitive impairment.

### 2.4. Additional Variables

This study also included data collection on participants’ educational level and the duration of HIV infection, as both factors are known to influence cognitive outcomes in PLWHA.

## 3. Statistical Analysis

All statistical analyses were conducted using MedCalc Statistical Software version 23.0.2 (MedCalc Software Ltd., Ostend, Belgium). Results are reported with corresponding *p*-values and 95% confidence intervals (CIs). Continuous variables are expressed as median and interquartile range (IQR) for non-normally distributed data and/or as percentages where appropriate. The correlation between the MoCA and the IHDS was evaluated using Spearman’s rank correlation. Following data collection, the study sample was randomly divided into a validation and a test sample using the random allocation function in MedCalc. In the validation sample, the performance of the IHDS in detecting cognitive impairment was evaluated using the area under the Receiver Operating Characteristic (ROC) curve. The Youden J statistics were applied to determine the optimal IHDS cut-off value for identifying cognitive impairment. Categorical variables in the test sample were compared using the Chi-square test, while comparisons of continuous variables between the validation and test samples were performed using the Mann–Whitney U test.

## 4. Results

### 4.1. Demographic, Clinical, Laboratory, and Cognitive Characteristics

The demographic, clinical, laboratory, and cognitive characteristics of the study population are summarized in Table 1. The sample comprised 65.6% male participants; all patients were on cART. The median age was 57 years (IQR: 51–60), with 88 individuals (30.0%) aged 60 years or older.

Regarding education, the median number of school years was 11 (IQR: 8–13). More specifically, 35.7% of participants had completed only lower secondary education, while 44.9% had achieved at least upper secondary education (≥13 years of schooling).

The median duration of HIV infection was 25 years (IQR: 13–31), with 76 participants (25.9%) having been living with HIV for more than 30 years.

Cognitive performance is detailed in Table 1 The median total MoCA score (adjusted for education) was 25 (IQR: 23–27); nine participants (3.1%) obtained the maximum score, while 24.5% scored below the cut-off of 23, of whom 68.0% were male. The median IHDS total score was 9.5 (IQR: 8–11), with forty-one participants (13.9%) achieving the highest score. The validation and test samples comprised 162 and 132 individuals, respectively. Detailed characteristics of each group are reported in Table 1. Within the validation sample, 28.4% of patients scored below 23 on the MoCA, compared to 19.7% in the test sample. No statistically significant differences were observed between the two samples based on the Mann–Whitney test.

### 4.2. Correlation Between IHDS and MoCA

The correlation coefficient (Spearman’s rho) between the IHDS and MoCA was 0.411 (*p* < 0.0001, 95% CI 0.311–0.501), indicating a moderate positive correlation between the two cognitive screening tools.

### 4.3. ROC Curve Analysis and Determination of IHDS Cut-Off

**Figure 1:**
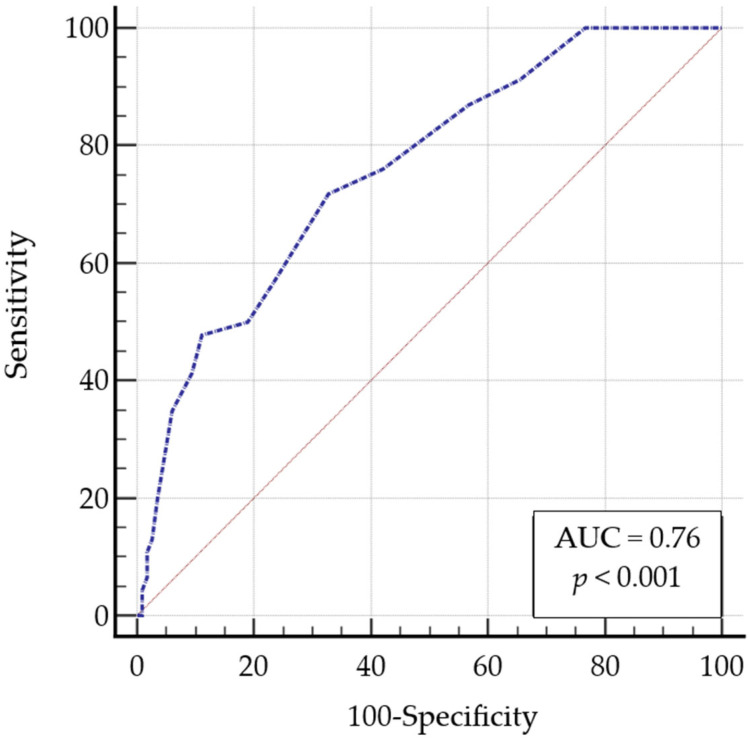
Area under the receiver operating characteristic curve for IHDS-IT. Abbreviation: AUC: Area Under the Curve; IHDS-IT, Italian International HIV Dementia Scale.

A ROC curve was constructed to determine the optimal IHDS-IT cut-off score for intercepting cognitive impairment in the validation sample. The MoCA, using a threshold score of 23, served as the classification variable. The ROC analysis yielded an Area Under the Curve (AUC) of 0.76 (95% CI 0.67–0.82) (Figure 1). An IHDS score of ≤9 was identified as the optimal cut-off (Table 2), with a positive likelihood ratio (+LR) of 2.19 and a negative likelihood ratio (–LR) of 0.42. At this threshold, sensitivity was 71.7% (95% CI: 56.5–84.0), and specificity was 67.2% (95% CI: 57.9–75.7). When the newly identified IHDS cut-off of 9 was applied to the test sample, 19 of the 26 participants (73.1%) with MoCA scores below 23 also scored abnormally on the IHDS. Conversely, among the 106 individuals with MoCA scores above 23, 58.5% had a normal IHDS score.

## 5. Discussion

While several screening tools, like the HIV Dementia Scale [40], have Italian validation available, the IHDS has not yet been formally adapted for Italian populations. In this framework, the current study aims to address this gap by adapting the IHDS for use in the Italian PLWHA, providing a standardized and accessible screening tool that can be easily implemented into routine clinical practice in Italy.

To achieve this objective, a sample of 294 HIV-positive patients underwent cognitive evaluation using the IHDS and MoCA as a reference test. Our analysis revealed a moderate positive correlation between the IHDS and MoCA (Spearman’s rho = 0.411). The assessments are non-overlapping due to the distinct items assessed in each tool. The IHDS predominantly evaluated motor speed, psychomotor skills, and memory recall, representing narrower cognitive function dimensions [30,41,42,43,44,45]. In contrast, the MoCA provides a more comprehensive assessment of cognitive domains, including short-term memory, attention, working memory, and executive functions, commonly affected in PLWHA [34,35,36,37,46].

The ROC analysis, using the MoCA as the “classification variable”, yielded a fair AUC (0.76). This analysis identified an IHDS cut-off score of ≤9 as optimal for intercepting cognitive impairment in our validation sample with a sensitivity of 71.74% and a specificity of 67.24%. Interestingly, 73.1% of PLWHA classified as cognitively impaired according to the MoCA (cut-off < 23) also scored ≤9 on the IHDS-IT. These findings suggest that, although the tools assess different cognitive domains, both are effective in identifying cognitive impairment in PLWHA. The effectiveness of using both tests as screening tools is emphasized by their combined impact, particularly since the IHDS requires less time to administer than the MoCA [44,45].

The IHDS cut-off score identified in our study is slightly lower than the threshold generally recommended in the literature. Indeed, a systematic review and meta-analysis by Rosca et al. (2021) [45] suggests an IHDS cut-off of 10 as the optimal threshold for HANDs detection. This recommendation was based on the pooled accuracy measures, showing that an IHDS score of 10 achieves a sensitivity of 61.9% and a specificity of 67.5%. However, the authors themselves acknowledged that studies included in the review were heterogeneous, especially in the group of symptomatic HANDs and HAD. In our study, a cut-off value of 10 yielded a higher sensitivity (86.96) but a lower specificity (43.10).

The difference between our ideal IHDS cut-off and the one proposed by Rosca et al. [45] may reflect specific characteristics of the HIV populations studied. These included demographic, clinical, and pharmacological differences. Notably, our sample had a median age of 57, with 30% of patients aged over 60. In contrast, the highest mean age reported in the review was 53 (11) years. Furthermore, the majority of PLWHA in our study had a suppressed viral load at the time of assessment, while many studies included in the review did not provide this information. All 294 patients in our sample were receiving cART, while only five of the studies included in the systematic review reported that at least 89% of participants were on cART. In the remaining studies, no information about therapy was provided, or a percentage below 80% of the participants were on ART. Additionally, our study’s prevalence of patients over 60 years old (30%) and those living with HIV for more than 30 years (25.9%) highlights the vulnerability of the aging HIV population to cognitive decline. Therefore, both HIV-specific factors and age-related neurodegenerative processes may influence this decline.

These findings also expand on our previously published study, which demonstrated the utility of both IHDS and MoCA in detecting cognitive impairment in PLWHA [25]. In that study, we adopted IHDS and MoCA cut-offs of ≤10 and ≤25, respectively, in line with the common practices. In the present study, the MoCA cut-off was lowered to 23 based on recent systematic evidence suggesting improved diagnostic performance at this threshold [35]. The identification of a lower IHDS threshold in this study may therefore reflect both the demographic and clinical profile of our sample, as well as the use of a more stringent MoCA criterion as reference. Importantly, both the present and previous studies support the combined use of MoCA and IHDS as complementary screening tools in HIV populations. Indeed, in light of the moderate overall AUC values observed in our study (0.76; 95% CI: 0.67–0.82), the IHDS appears useful but should always be interpreted in the context of other screening tools or comprehensive neuropsychological assessments.

However, the use of a lower IHDS threshold raises important clinical considerations. A cut-off of ≤9, while improving specificity, may reduce sensitivity and increase the risk of false negatives, potentially missing milder forms of cognitive impairment. This is particularly relevant in clinical contexts where early detection is critical. Further research is needed to establish the most appropriate balance between sensitivity and specificity across different HIV-positive populations, particularly in older adults, individuals with well-controlled infections, and those in outpatient care settings, where subtle cognitive changes may go unnoticed.

This study has several limitations. Its cross-sectional design precludes conclusions about temporal or causal associations. Moreover, the lack of a comprehensive neuropsychological battery, the gold standard for HANDs diagnosis, limits the validation of the IHDS-IT. Although MoCA served as a comparator for ROC analysis, its use as a screening tool rather than a diagnostic reference may have affected the accuracy estimates. Finally, the single-center setting and inclusion of well-managed patients may limit the generalizability of our findings to broader or more diverse HIV populations. Future longitudinal studies are warranted to confirm these findings and to assess key outcomes over time, such as the progression of cognitive impairment and the predictive validity of the IHDS-IT.

## 6. Conclusions

Our study suggests that IHDS-IT is a potentially reliable screening tool for cognitive impairment in clinical practice, as it has discrete sensitivity and specificity. The performance of the IHDS-IT supports its application in combination with the MoCA, providing a balanced approach: the IHDS-IT offers a rapid, time-efficient screening option, while the MoCA provides a more comprehensive evaluation of cognitive domains.

## Figures and Tables

**Table 1:** (**A**) Demographic, clinical, and laboratory characteristics of the sample. (**B**) Cognitive performance of the sample on MoCA and IHDS.

(**A**)			
**Variables**	**All** **(*n* = 294)**	**Validation Sample** **(*n* = 162)**	**Test Sample** **(*n* = 132)**
**Sex, Male—*n* (%)**	193 (65.6%)	104 (64.2%)	89 (67.4%)
**Age (years)**	57 (51–60)	57 (50–60)	57 (52–61)
**Education (years)**	11 (8–13)	11.5 (8–13)	11 (8–13)
**Years of HIV infection**	25 (13–31)	25 (13–30)	25.5 (11.5–31.0)
**HIV-RNA (copies/µL)**	0 (0–0)	0 (0–0)	0 (0–0)
**HIV-RNA Zenith (copies/µL)**	67,357 (16,616–166,400)	67,357 (16,616–166,400)	67,357 (16,616–166,400)
**CD4+ T-lymphocytes (cells/µL)**	742 (534–985)	784.5 (552–1003)	702.5 (483.5–958.5)
**CD4+ T-lymphocyte Nadir (cells/µL)**	250.5 (118–389)	249.5 (125–386)	253.5 (112.5–395.0)
**CD4+/CD8+ T-lymphocyte ratio**	1.04 (0.65–1.74)	1.03 (0.65–1.68)	1.07 (0.67–1.97)
(**B**)			
**Variables**	**All** **(*n* = 294)**	**Validation Sample** **(*n* = 162)**	**Test Sample** **(*n* = 132)**
**MoCA-Total Score (adj. education)**	25 (23–27)	25 (22–27)	25 (23–27)
**MoCA-Visuospatial/Executive**	4 (3–5)	4 (3–5)	4 (3–5)
**MoCA-Naming**	3 (3–3)	3 (3–3)	3 (3–3)
**MoCA-Attention**	5 (4–6)	5 (4–6)	5 (5–6)
**MoCA-Language**	2 (2–3)	2 (2–3)	2 (2–3)
**MoCA-Abstraction**	2 (2–2)	2 (2–2)	2 (1.5–2.0)
**MoCA-Delayed Recall**	2 (1–4)	2 (1–4)	2 (1–4)
**MoCA-Orientation**	6 (6–6)	6 (6–6)	6 (6–6)
**IHDS-Total Score**	9.5 (8–11)	9.5 (8–11)	9 (8–11)
**IHDS-Recall**	3 (2–4)	3 (2–4)	3 (2–4)
**IHDS-Motor Speed**	4 (2–4)	4 (2–4)	4 (2–4)
**IHDS-Psychomotor Speed**	4 (3–4)	4 (3–4)	4 (3–4)

Data are presented as median (interquartile range) or number (percentage). Abbreviations: adj. education, education-adjusted; HIV, human immunodeficiency virus; IHDS, International HIV Dementia Scale; MoCA, Montreal Cognitive Assessment.

**Table 2:** ROC analysis: sensitivity, specificity, and likelihood ratios for IHDS-IT scores in intercepting cognitive impairment (reference: MoCA < 23).

IHDS-IT Score	Sensitivity	95% CI	Specificity	95% CI	+LR	−LR
<1	0.00	0.0–7.7	100.00	96.9–100.0	/	1.00
≤1	0.00	0.0–7.7	99.14	95.3–100.0	0.00	1.01
≤2	2.17	0.06–11.5	99.14	95.3–100.0	2.52	0.99
≤4	4.35	0.5–14.8	99.14	95.3–100.0	5.04	0.96
≤4.5	6.52	1.4–17.9	98.28	93.9–99.8	3.78	0.95
≤5	10.87	3.6–23.6	98.28	93.9–99.8	6.30	0.91
≤5.5	13.04	4.9–26.3	97.41	92.6–99.5	5.04	0.89
≤6	19.57	9.4–33.9	96.55	91.4–99.1	5.67	0.83
≤6.5	34.78	21.4–50.2	93.97	88.0–97.5	5.76	0.69
≤7	41.30	27.0–56.8	90.52	83.7–95.2	4.36	0.65
≤7.5	47.83	32.9–63.1	88.79	81.6–93.9	4.27	0.59
≤8	50.00	34.9–65.1	81.03	72.7–87.7	2.64	0.62
≤8.5	56.52	41.1–71.1	76.72	68.0–84.1	2.43	0.57
**≤9**	**71.74**	**56.5–84.0**	**67.24**	**57.9–75.7**	**2.19**	**0.42**
≤9.5	76.09	61.2–87.4	57.76	48.2–66.9	1.80	0.41
≤10	86.96	73.7–95.1	43.10	33.9–52.6	1.53	0.30
≤10.5	91.30	79.2–97.6	34.48	25.9–43.9	1.39	0.25
≤11	100.00	92.3–100.0	23.28	15.9–32.0	1.30	0.00
≤11.5	100.00	92.3–100.0	18.10	11.6–26.3	1.22	0.00
≤12	100.00	92.3–100.0	0.00	0.0–3.1	1.00	

The bold row indicates the optimal IHDS-IT cut-off score (≤9), as determined by ROC analysis. Abbreviations: +LR, positive likelihood ratio; −LR, negative likelihood ratio; CI, confidence interval; ROC, Receiver Operating Characteristic.

## Data Availability

The data and materials used and/or analyzed during the current study are not publicly available. They are available from the corresponding author upon reasonable request.

## Abbreviations

The following abbreviations are used in this manuscript:

AIDS	Acquired Immunodeficiency Syndrome
AUC	area under the ROC curve
cART	Combination Antiretroviral Therapy
CNS	Central Nervous System
CPE	CNS penetration effectiveness
HANDs	HIV-associated neurocognitive disorders
HIV	Human Immunodeficiency Virus
HAD	HIV-associated dementia
IHDS	International HIV Dementia Scale
IHDS-IT	Italian International HIV Dementia Scale
IQR	interquartile range
MoCA	Montreal Cognitive Assessment
−LR	negative likelihood ratio
PLWHA	people living with HIV/AIDS
+LR	positive likelihood ratio
ROC	Receiver Operating Characteristic
95%CI	95% confidence interval
